# Association between physical activity and mental health among Yi students aged 10–18 years in Southwest China: a school-based cross-sectional study

**DOI:** 10.3389/fpubh.2026.1878144

**Published:** 2026-06-24

**Authors:** Qingju Shao, Xiaolin Zhang, Xuefang Li, Jiaheng Wang

**Affiliations:** 1 School of Physical Education, Sichuan Normal University, Chengdu, China; 2 School of Marxism, Chongqing Normal University, Chongqing, China

**Keywords:** loneliness, mental health, physical activity, school health, social anxiety, Yi students

## Abstract

**Background:**

Physical activity is a modifiable behavior that may contribute to youth mental health, yet evidence from ethnic minority school populations in Southwest China remains limited.

**Methods:**

This school-based cross-sectional study analyzed questionnaire data from 671 Yi students aged 10–18 years. Physical activity was assessed using an 8-item PAQ-like measure. Mental health was operationalized through three adverse domains: anxiety, loneliness, and social anxiety. Age, sex, and school stage were included as covariates. Psychometric performance was examined using Cronbach’s alpha, the Kaiser–Meyer–Olkin statistic, and Bartlett’s test of sphericity. Primary associations were estimated with ordinary least squares regression using HC3 robust standard errors, followed by sex- and stage-stratified models, PAQ tertile analyses, linear trend tests, strict complete-item sensitivity analyses, and exploratory parenting extension models.

**Results:**

The final sample included 379 girls and 292 boys with a mean age of 13.87 years. Reliability was acceptable to excellent across the four core scales (alpha = 0.709–0.938), and factorability indices supported the use of scores in this sample. Physical activity was inversely correlated with loneliness (*r* = −0.114, *p* = 0.003), but not with anxiety or social anxiety. After adjustment, higher physical activity remained associated with lower loneliness (*B* = −2.13, 95% CI −3.40 to −0.85, *p* = 0.001), whereas associations with anxiety and social anxiety were not significant. Follow-up analyses showed that the inverse PAQ-loneliness association was more evident among boys and among upper primary and junior secondary students, and the loneliness gradient across PAQ tertiles was statistically significant. In exploratory contextual models, the PAQ coefficient attenuated after father- and mother-related parenting composites were added, while father support and father harsh control showed clear associations with loneliness.

**Conclusion:**

In this minority school sample, the mental health pattern associated with physical activity was selective rather than global. Higher physical activity was most consistently linked to lower loneliness, while its associations with anxiety and social anxiety were not robust. These findings support a more contextualized public health interpretation of youth physical activity, in which relational connectedness and family climate may shape psychological benefits.

## 1 Introduction

Adolescence is a sensitive developmental period in which social role transition, emotional regulation, and health behavior formation occur simultaneously, making it a critical window for research on modifiable determinants of wellbeing (1). In public health terms, the importance of adolescent health is not limited to the present; it also influences later adult functioning and the health of the next generation. For that reason, behaviors that can be promoted in school settings are particularly attractive targets for population-level prevention. Globally, physical activity declines across adolescence, while internalizing symptoms become more prominent in school populations. This co-occurrence has intensified interest in whether movement-related behaviors can serve as feasible targets for mental health promotion in routine educational settings. The appeal of this approach is practical as well as theoretical: unlike specialized therapy services, school-based activity opportunities can reach large numbers of students, including those in areas where mental health resources remain limited. Physical activity is one of the most frequently discussed candidate behaviors because it is linked not only to physical fitness and cardiometabolic health but also to broader developmental and mental health benefits for young people (2). Recent review and meta-analytic evidence support the broad proposition that physical activity can be related to better mental health in children and adolescents, although the strength and consistency of that relationship vary by outcome, population, study design, and intervention context (3). Mechanistic and moderation-focused evidence further suggests that the mental health relevance of physical activity is shaped by psychosocial factors such as self-esteem, self-efficacy, resilience, social support, and social connection rather than by movement dose alone (4).

However, the association between physical activity and mental health should not be treated as universally uniform. Recent syntheses of intervention studies indicate that exercise-based programs can reduce depression, anxiety, and psychological distress, but effect sizes are heterogeneous and depend on intervention form, duration, intensity, and population characteristics (5). A more recent review focusing on healthy adolescents reached a similar conclusion, arguing that mental health benefits are present but uneven across symptom domains and study contexts (6). Large multi-country adolescent data have likewise linked higher physical activity with lower anxiety and better wellbeing (7). School-based intervention evidence also shows promise, but not every intervention improves all psychological endpoints to the same degree (8). Among the adverse domains relevant to adolescence, loneliness deserves special attention. Loneliness is not simply an unpleasant feeling; it is a social-cognitive experience that has been linked to broad mental health difficulties in youth and to poorer long-term outcomes if sustained over time (9). Across the life course, loneliness and social isolation are also associated with elevated morbidity and mortality risk, underscoring that social disconnection is a substantive public health concern rather than a minor emotional nuisance (10).

At the same time, loneliness should not be conflated with anxiety or social anxiety. Meta-analytic evidence indicates that loneliness and social anxiety are meaningfully related but remain distinct constructs across childhood and adolescence (11). This distinction matters because adolescents can feel lonely without exhibiting a strong fear of evaluation, and conversely, they can experience marked social anxiety while still being socially embedded. General anxiety is similarly multidimensional, and the commonly used youth anxiety measures are designed to capture several symptom clusters rather than a single social process (12). Social anxiety is especially relevant in school populations because it is closely tied to peer evaluation, self-presentation concerns, and perceived belonging. Earlier studies on adolescent social anxiety demonstrated that fear in peer contexts is strongly shaped by friendship quality and peer relations (13). Subsequent conceptual and etiological models have emphasized the importance of unfamiliar peer situations, anticipated evaluation, and avoidance processes in the development and maintenance of social anxiety symptoms (14, 15). More recent meta-analytic findings show that poor peer functioning prospectively predicts higher social anxiety in adolescence, reinforcing that the social environment is central to interpretation (16). Population-based adolescent data further show that social anxiety varies with poor self-rated mental and physical health (17).

Recent studies from China have begun to examine the relation between physical activity and social anxiety more directly, but the evidence remains mixed and context-specific. Longitudinal research has suggested that physical exercise may be associated with lower adolescent social anxiety through psychological resilience (18). Additional cross-sectional work in Chinese adolescents has connected moderate-to-vigorous physical activity, self-disclosure, and social avoidance to social anxiety (19). Other Chinese studies have implicated self-esteem in the physical activity-social anxiety link (20), while separate analyses have highlighted social support and psychological capital (21). Body image and chained mediation models have also been proposed, although most of these studies have been conducted among university students rather than younger or culturally distinctive samples (22, 23). An important implication of this literature is that physical activity may be more relevant to some psychological domains than to others. Outcomes that are tightly bound to peer inclusion, belonging, and relational experience may be more sensitive to everyday activity patterns than outcomes rooted in generalized worry or trait-like threat sensitivity. For school-based public health research, it is therefore useful to model loneliness, anxiety, and social anxiety separately rather than assuming that they all respond in the same way to high activity.

The broader daily-life context is also important. Physical activity coexists with sleep, screen exposure, academic demands, and internet use, and these factors may jointly shape anxiety-related outcomes in adolescents (24). Longitudinal evidence suggests that movement and sedentary behavior are bidirectionally related to internalizing symptoms over time, implying that cross-sectional associations may be weaker or less consistent than simple common-sense expectations suggest (25). Studies from less-developed areas of China also link physical activity and screen-related sedentary behavior to adolescent anxiety (26). Brazilian adolescent datasets further indicate that activity and sedentary behavior are associated with social isolation or connectedness-related outcomes, which is relevant when loneliness is treated as a distinct endpoint (27, 28). Recent syntheses further suggest that the context in which physical activity occurs—structured sport, school physical education, activity breaks, or informal self-directed movement—may shape whether activity is psychologically beneficial, and that intervention characteristics and social conditions are important for interpreting youth mental health outcomes (29, 30).

These issues become even more important in understudied populations. In Southwest China, Yi students constitute a culturally distinctive population whose school health experiences are unlikely to be fully represented by evidence from metropolitan Han-majority samples or Western cohorts. A recent study among Yi primary school students linked physical activity to academic burnout through psychosocial pathways (31), but direct evidence of physical activity and multiple adverse mental health domains in Yi youth remains scarce. The present study, therefore, examined whether physical activity was associated with anxiety, loneliness, and social anxiety among Yi students aged 10–18 years in Southwest China, and whether any observed association remained stable across sex, school stage, and exploratory family-context models.

## 2 Materials and methods

### 2.1 Study design and participants

This school-based cross-sectional study used paper-based questionnaires administered in schools in Southwest China. The original workbook contained 712 records after deletion of a non-data header row. Because the substantive aim of the study was to focus on Yi youth rather than on ethnic-group comparison, ethnicity was treated as an inclusion criterion rather than an analytical covariate. We restricted the target population to students identified as Yi and aged 10–18 years. After excluding records outside the target age range and cases with missing core exposure or outcome data, the final analysis-ready sample comprised 671 participants.

The age restriction was conceptually motivated rather than merely data-driven. Ages 10–18 years capture late childhood through adolescence in a school context, covering upper primary, junior secondary, and senior secondary stages while excluding sparse older observations that would have introduced additional heterogeneity. This framing also aligns with the article’s public health emphasis on school-aged adolescents and early life-course prevention. The reporting structure follows the STROBE recommendations for observational studies (32, 33).

### 2.2 Measures

Physical activity was assessed with an 8-item PAQ-like child questionnaire administered the previous 7 days. The instrument combined one checklist-style activity-frequency item with seven items covering physical education participation, midday activity, after-school activity, evening activity, weekend activity, overall weekly activity, and day-specific activity frequency. Responses were harmonized in a common 1–5 direction so that higher values reflected greater habitual activity. Because the local instrument followed the response logic of the PAQ literature but was not in the verbatim original PAQ-C form, we refer to it as PAQ-like rather than as a direct administration of the original validated questionnaire (34, 35). A clean mean PAQ score was calculated when at least seven of the eight items were valid.

Mental health was operationalized through three adverse domains rather than a single undifferentiated total. Anxiety symptoms were assessed with a 41-item child self-report measure conceptually consistent with the multidimensional framework proposed by Spence (12). Loneliness was assessed using a revised UCLA-based adolescent measure; item directions were carefully checked, and reverse-coded where necessary so that higher scores consistently indicated greater loneliness (36, 37). Social anxiety was assessed with a 10-item child / adolescent measure focusing on fear of peer evaluation, shyness in unfamiliar peer settings, and social avoidance, which maps well onto the adolescent peer-relations literature (13, 14).

Age, sex, and school stage were included as covariates in all primary adjusted models. School stage was derived from grade and categorized as upper primary, junior secondary, or senior secondary. Because the questionnaire package also contained father- and mother-related parenting items, exploratory contextual models were later fitted to evaluate whether the primary PA-loneliness association was attenuated after family-climate variables were considered.

### 2.3 Data cleaning and score construction

Data cleaning was performed before any inferential analysis. First, impossible or out-of-range responses were set to missing. Second, grade labels were standardized to derive a consistent school-stage variable. Third, age-grade inconsistencies were reviewed, and records outside the target age range were excluded. Fourth, the loneliness scale was rescored after checking item direction against questionnaire wording, because the raw workbook score could not be treated as analysis-ready. Fifth, de-identified participant IDs were assigned, and the cleaned workbook was transformed into both Excel- and SPSS-compatible analysis files.

### 2.4 Psychometric evaluation

Missing-data handling was intentionally conservative. Participants were retained only when the exposure and outcome variables required for the main analyses were available after cleaning, and scale totals were computed only when the predefined minimum number of valid items had been met. This approach prioritized comparability across models and reduced the risk of artificial score inflation or excessive prorating distorting associations.

Because all four core instruments had already been used in prior research, the psychometric evaluation in the present study was designed to support the use of scores in this specific sample rather than redevelop the scales. Internal consistency was estimated using Cronbach’s alpha. In addition, sample-based structural validity support was examined using the Kaiser–Meyer–Olkin (KMO) statistic and Bartlett’s test of sphericity. This strategy was chosen to provide evidence that the item sets were sufficiently coherent and factorable for score use in the current sample, while acknowledging that full-scale redevelopment or multi-group confirmatory modeling was beyond the scope of a school-based epidemiologic analysis. No validated categorical cutoffs were assumed for the anxiety, loneliness, or social anxiety scales in this sample; accordingly, score means were interpreted descriptively rather than as diagnostic severity levels.

### 2.5 Statistical analysis

Descriptive statistics were calculated for participant characteristics and for the four core scales. Pearson’s correlations were used to estimate bivariate associations among physical activity and the three mental health outcomes. Primary inferential analyses used ordinary least square regression with HC3 robust standard errors, which was selected to reduce sensitivity to heteroscedasticity in observational school data. Separate adjusted models were fitted for anxiety, loneliness, and social anxiety, controlling for age, sex, and school stage.

To evaluate heterogeneity and robustness, we fitted sex-stratified and school stage-stratified models, compared PAQ tertiles, tested linear trends across PAQ tertiles, and reran the primary models in a strict complete-item dataset. Interaction terms were explored to assess whether the PAQ coefficients differed materially across subgroups. Finally, because the questionnaire package contained extensive father- and mother-related items, exploratory parenting composites were generated and entered into loneliness models as contextual extension variables. These extension models were explicitly treated as exploratory because the composites were content-derived from item semantics.

## 3 Results

### 3.1 Sample characteristics and psychometric performance

The final analytical sample included 671 Yi students. Girls accounted for 56.5% of the sample and boys for 43.5%. Mean age was 13.87 years (SD = 1.64). With respect to school stage, 38.5% of students were in upper primary school, 54.4% were in junior secondary school, and 7.0% were in senior secondary school. This composition indicates that the study predominantly captured late-childhood and early-to-mid-adolescent school experiences rather than late-adolescent post-compulsory trajectories.

The psychometric results support the use of scores in the present sample. Cronbach’s alpha was 0.709 for PAQ, 0.938 for anxiety, 0.735 for loneliness, and 0.807 for social anxiety. These values indicated acceptable internal consistency for the activity and loneliness scales and strong consistency for the anxiety-related scales. KMO values ranged from 0.809 to 0.948, and Bartlett’s tests were significant for all scales, suggesting that the item sets showed adequate sampling adequacy and factorability. In the context of an observational public health analysis using previously established measures, these results were considered sufficient to justify using the cleaned total scores.

The psychometric pattern is worth emphasizing because the analysis depended on the score quality. The PAQ alpha slightly above 0.70 is typical of brief youth activity measures used in heterogeneous school samples, where activity varies by season, class schedule, and opportunity. The very high alpha for the anxiety scale reflects the breadth and internal coherence of the symptom inventory, whereas the more moderate alpha for loneliness is consistent with the scale’s mix of direct and reverse-worded interpersonal items.

Descriptive statistics further suggested that the three adverse mental health domains should not be treated interchangeably. The mean anxiety score was 22.48 (SD = 14.75), mean loneliness score was 40.80 (SD = 9.22), and mean social anxiety score was 6.47 (SD = 4.36). For interpretive context, the PAQ-like index ranged from 1 to 5, so the sample mean of 2.26 indicated generally low-to-moderate weekly activity. The possible score ranges were 0–82 for anxiety, 16–80 for loneliness, and 10–30 for social anxiety. Because validated categorical cutoffs were not available for these measures in this school-based sample, the means are best understood descriptively and comparatively rather than at high, moderate, or low clinical levels (Tables 1, 2).

**Table 1:** Sample characteristics of the final analytical sample.

Characteristic	Value
Final analytical sample, *n*	671
Girls, *n* (%)	379 (56.5%)
Boys, *n* (%)	292 (43.5%)
Age, mean ± SD, years	13.87 ± 1.64
Upper primary, *n* (%)	258 (38.5%)
Junior secondary, *n* (%)	365 (54.4%)
Senior secondary, *n* (%)	47 (7.0%)

Percentages were calculated from the final analysis-ready sample (*n* = 671).

**Table 2:** Descriptive statistics, reliability, and sample-based structural validity of the four core scales.

Scale	Items	*N*	Mean ± SD	Cronbach’s *α*	KMO	Bartlett *χ*^2^ (df)	*p*
PAQ	8	671	2.26 ± 0.57	0.709	0.809	862.74 (28)	<0.001
Anxiety	41	671	22.48 ± 14.75	0.938	0.948	9,192.55 (820)	<0.001
Loneliness	16	671	40.80 ± 9.22	0.735	0.888	2,723.65 (120)	<0.001
Social anxiety	10	671	6.47 ± 4.36	0.807	0.882	1,434.39 (45)	<0.001

KMO = Kaiser–Meyer–Olkin statistic. Bartlett’s tests were significant for all scales. Possible score ranges were 1–5 for PAQ, 0–82 for anxiety, 16–80 for loneliness, and 10–30 for social anxiety. Higher scores indicate more physical activity (PAQ) or greater symptom burden (mental health scales). No validated categorical cutoffs were applied; means are interpreted descriptively.

### 3.2 Bivariate correlations and primary adjusted models

**Figure 1:**
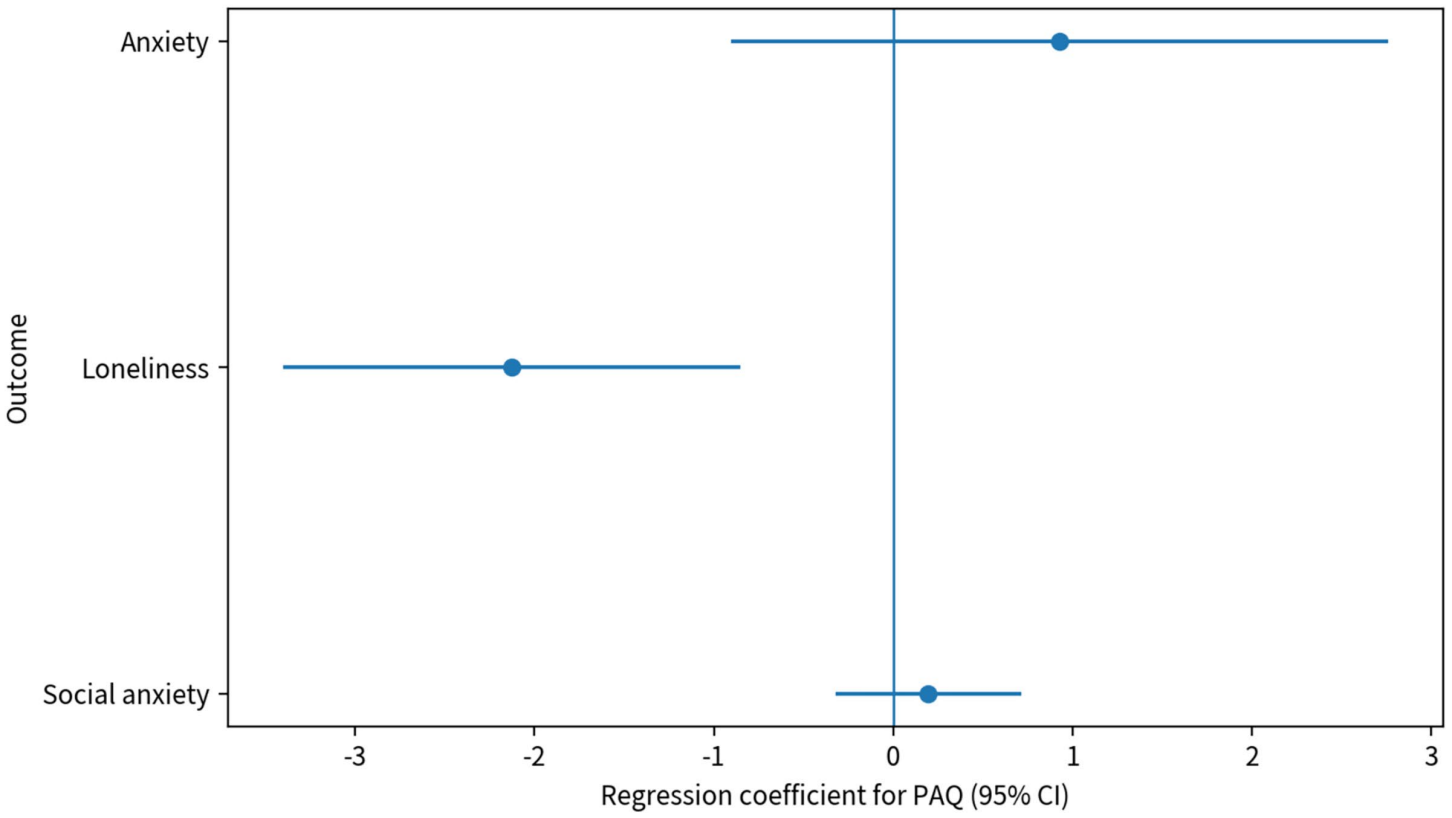
Main adjusted associations between PAQ and the three outcomes. Error bars represent 95% confidence intervals.

As shown in Table 3, physical activity was inversely related only to loneliness, whereas its correlations with anxiety and social anxiety were not statistically significant. This pattern is important because it shows that, even before adjustment, the observable mental health signal linked to activity was concentrated in one domain rather than distributed evenly across all three outcomes. The adjusted regression models led to the same substantive conclusion. After controlling for age, sex, and school stage, the coefficient for PAQ remained significant only in the loneliness model (*B* = −2.13, 95% CI −3.40 to −0.85, standardized beta = −0.132, *p* = 0.001). The corresponding coefficients for anxiety (*B* = 0.93, *p* = 0.321) and social anxiety (*B* = 0.20, *p* = 0.459) were small and not significant. Although the effect size for loneliness was modest, its direction was stable, and its confidence interval did not cross null, which made it the clear primary finding of the study. The main adjusted coefficients and confidence intervals are visualized in Figure 1.

**Table 3:** Pearson’s correlation matrix for the core study variables (lower triangle shown).

Variable	PAQ	Anxiety	Loneliness	Social anxiety
PAQ	1.000	–	–	–
Anxiety	0.042	1.000	–	–
Loneliness	−0.114	0.310	1.000	–
Social anxiety	0.034	0.610	0.347	1.000

Lower triangle coefficients are displayed to avoid duplication. Higher scores indicate more physical activity or more severe adverse mental health symptoms, respectively.

A one-point increase in PAQ was associated with a little more than a two-point decrease in loneliness score after adjustment. This should not be interpreted as a large clinical effect. Rather, it indicates a modest but reliable population-level association, which may still matter in school public health planning when exposure is common, modifiable, and relatively low-cost to promote. Equally important, the current data do not support the broad claim that physical activity uniformly improves mental health. In this sample, the evidence instead supports a selective interpretation: greater physical activity was associated with lower loneliness, whereas adjusted associations with total anxiety and social anxiety were not robust (Table 4).

**Table 4:** Adjusted associations between physical activity and the three mental health outcomes.

Outcome	*N*	Adjusted B for PAQ	95% CI	Standardized *β*	*p*	Model *R*^2^
Anxiety	670	0.93	−0.90 to 2.76	0.036	0.321	0.084
Loneliness	670	−2.13	−3.40 to −0.85	−0.132	0.001	0.071
Social anxiety	670	0.20	−0.32 to 0.71	0.027	0.459	0.065

Models adjusted for age, sex, and school stage; HC3 robust standard errors were used.

### 3.3 Heterogeneity and robustness

Background group comparisons suggested modest heterogeneity in the sample. Boys reported slightly higher PAQ scores than girls, and girls reported somewhat higher anxiety scores. Loneliness was marginally higher among boys, whereas social anxiety showed no clear sex difference in unadjusted comparisons. Across school stages, the most notable differences involved social anxiety and overall activity distribution rather than a monotonic worsening of every outcome with educational progression (Table 5).

**Table 5:** Follow-up analyses for heterogeneity and robustness.

Analysis	Outcome	*B*	95% CI	*p*
Sex-stratified (male)	Loneliness	−2.73	−4.53 to −0.93	0.003
Sex-stratified (female)	Loneliness	−1.44	−3.25 to 0.37	0.119
Stage-stratified (upper primary)	Loneliness	−2.23	−4.18 to −0.28	0.025
Stage-stratified (junior secondary)	Loneliness	−2.02	−3.81 to −0.23	0.027
Stage-stratified (senior secondary)	Loneliness	−2.94	−8.81 to 2.92	0.325
PAQ tertiles (medium vs. low)	Loneliness	−1.80	−3.49 to −0.10	0.038
PAQ tertiles (high vs. low)	Loneliness	−2.60	−4.26 to −0.94	0.002
Per tertile trend	Loneliness	−1.30	−2.13 to −0.47	0.002
Sensitivity (strict complete-item sample)	Loneliness	−2.07	−3.43 to −0.71	0.003
Interaction: PAQ × senior secondary vs. upper primary	Social anxiety	−2.91	−4.89 to −0.93	0.004

The stage-specific social anxiety interaction should be interpreted as exploratory because the senior secondary subgroup was small.

**Figure 2:**
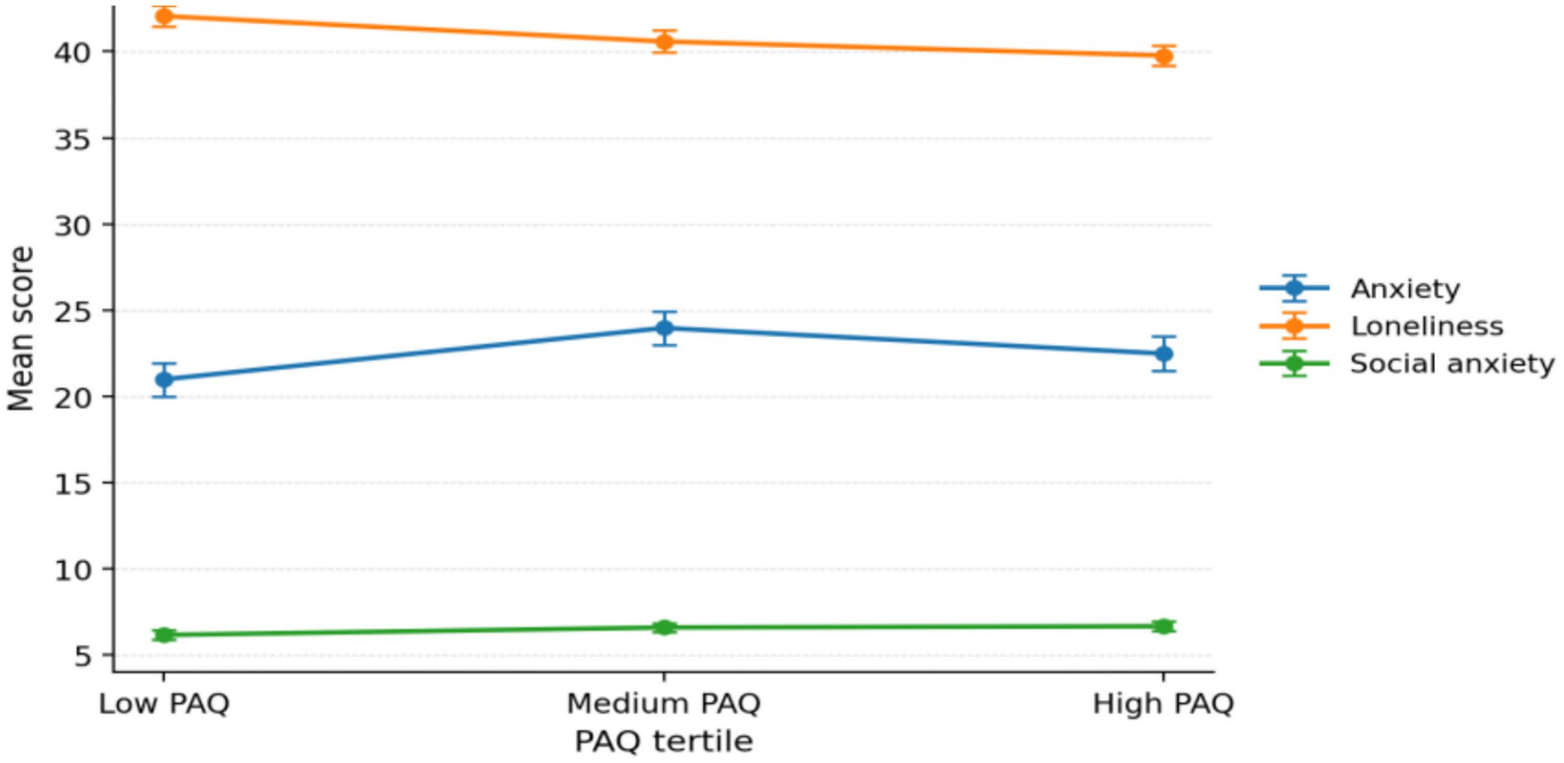
Mean outcome scores across PAQ tertiles. Error bars represent standard errors.

**Figure 3:**
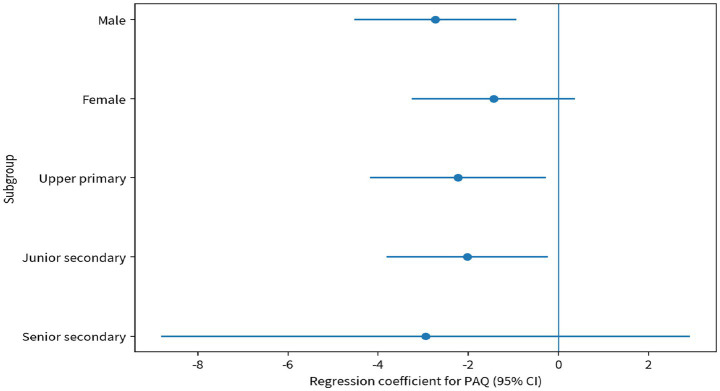
Adjusted PAQ-loneliness associations by subgroup. Error bars represent 95% confidence intervals.

The stratified models suggested that the inverse PAQ-loneliness association was not perfectly uniform. The coefficient remained significant among boys but not among girls. By school stage, the same inverse association was evident in upper primary and junior secondary students, but not in the smaller senior secondary subgroup. Because the senior secondary subsample was much smaller than the other two groups, the null estimate should be interpreted with caution; it may reflect stage-specific attenuation, limited power, or both. Robustness analyses strengthened confidence in the loneliness finding. Compared with the lowest PAQ tertile, both the middle and high tertiles showed lower loneliness; the linear trend across tertiles was significant, and the strict complete-item sensitivity analysis produced a similarly directed coefficient. By contrast, anxiety and social anxiety remained inconsistent across these supplementary analyses. Mean outcome scores across PAQ tertiles are presented in Figure 2, and subgroup-specific PAQ-loneliness coefficients are shown in Figure 3.

### 3.4 Exploratory parenting extension models

The parenting extension models provided a contextual test of whether the main loneliness association was independent of family relational climate. In the base loneliness model, the PAQ coefficient was −2.13 (*p* = 0.001), and the model R-squared was 0.071. After support-related parenting composites were entered, the PAQ coefficient weakened to −1.05 (*p* = 0.113), while father support showed a clear inverse association with loneliness than mother support. In the negative-only model, the PAQ coefficient remained significant but was somewhat smaller than in the base model, and father’s harsh control was positively associated with loneliness (Table 6).

**Table 6:** Exploratory parenting extension models for loneliness.

Model	*N*	PAQ *B*	PAQ *p*	Father support *B*	Father support *p*	Mother support *B*	Mother support *p*	Father negative *B*	Father negative *p*	Model *R*^2^
Base model	670	−2.13	0.001	–	–	–	–	–	–	0.071
Support-only extension	622	−1.05	0.113	−3.04	0.010	−2.04	0.073	–	–	0.134
Negative-only extension	622	−2.06	0.001	–	–	–	–	3.62	0.005	0.125
Full parenting extension	622	−1.12	0.071	−4.00	0.003	−2.45	0.056	4.41	<0.001	0.241

Parenting composites were content-derived and should be interpreted as exploratory contextual variables rather than official published subscales.

In the full parenting model, the model R-squared increased to 0.241. The PAQ coefficient attenuated further and no longer met the conventional significance threshold (B = −1.12, *p* = 0.071), whereas father support, father harsh control, and mother harsh control remained salient. These results should not be interpreted as evidence of mediation or causal overshadowing. Because the parenting composites were content-derived rather than officially validated subscales, they are best viewed as exploratory contextual indicators suggesting that the PAQ-loneliness association was embedded in a broader family climate.

## 4 Discussion

A further contribution to the study is methodological. Instead of treating mental health as a single omnibus outcome, the analysis separated anxiety, loneliness, and social anxiety, then tested whether the apparent activity signal survived adjustment and robustness checks. That strategy matters because studies that collapse across psychological domains may produce interpretations more global than the data warrant.

### 4.1 Principal findings

The present study examined whether physical activity was associated with three adverse mental health domains among Yi students aged 10–18 years in Southwest China. The principal finding was not that physical activity showed broad protective associations across all outcomes, but that it demonstrated a modest and comparatively stable inverse association with loneliness. The same pattern did not extend to adjusted models of total anxiety or social anxiety. This selective profile is consistent with the view that the relationship between physical activity and youth mental health may be meaningful but domain-specific rather than uniformly distributed across all forms of distress. From a public health perspective, that distinction matters because different psychological domains may reflect different mechanisms and contexts. In the current sample, activity was more closely linked to social-relational distress than to generalized anxiety symptoms.

### 4.2 Why loneliness shows the clear association?

The loneliness finding is theoretically plausible because much school-age physical activity is socially embedded. Students may be active while walking with peers, playing informal games, participating in physical education, or joining organized sports. These settings create repeated opportunities for co-presence, shared routines, and belonging. Contemporary loneliness scholarship emphasizes that loneliness is shaped not only by objective isolation but also by perceived connectedness and the quality of relational participation (38). In that sense, physical activity may relate to lower loneliness less through exercise dose alone than through the social world in which activity is often performed.

The social context of activity likely matters as well. A recent systematic review of intervention studies concluded that physical activity interventions can also alleviate loneliness and that improved social experiences may be one reason for this benefit (39). Organized sports participation among children and adolescents also appears to be associated with social and emotional outcomes (40). Although the present dataset did not distinguish between team and individual activity, the stronger and more consistent loneliness result aligns with the broader idea that the relational features of activity may be especially consequential. This interpretation should remain cautious because the current design cannot establish whether higher activity reduced loneliness, whether less-lonely students were more willing to participate, or whether both processes operated simultaneously.

### 4.3 Why did anxiety and social anxiety not show robust adjusted associations?

The absence of robust adjusted associations for anxiety and social anxiety should not be dismissed as implausible. Generalized anxiety is a broad construct influenced by worry, physiological arousal, academic pressure, sleep, and digital behavior. A self-reported activity index may therefore be too distal to show a stable linear relation with a heterogeneous total anxiety score in a single cross-sectional snapshot. Exercise-based anxiety reduction is often clear in intervention studies or review evidence where dose, adherence, population risk, and symptom change can be tracked more precisely (5, 41). Social anxiety may be even more context-dependent, because fear of evaluation, self-focused attention, and avoidance are not automatically modified by greater routine activity volume (15). Participation in physical activity may help some adolescents feel more socially integrated, but it may not directly reduce concern about negative evaluation, especially in performance-focused settings.

For minority school populations, contextual sensitivity is especially important. Cultural expectations, school transition, family climate, and differential access to organized sport can all shape what higher activity actually means in daily life. Reverse causation and bidirectional relationships are also plausible: students with lower loneliness or better overall adjustment may be more willing to participate in physical activity, just as activity may contribute to connectedness over time (42, 43). Recent syntheses of youth sport participation similarly indicate social and psychological benefits overall, but also substantial heterogeneity across sport type, social setting, developmental stage, and study design, meaning that null associations for some outcomes in some samples are not inherently contradictory to the broader literature (44).

### 4.4 Contextual interpretation: subgroup and parenting findings

The subgroup analyses add nuance rather than a second headline. The inverse PAQ-loneliness association was clearer among boys, upper primary students, and junior secondary students than among girls or senior secondary students, although the evidence for interaction was not uniformly strong. This pattern may reflect real developmental differences in how activity functions socially across school stages, but it may also partly reflect small subgroup sizes and different participation contexts. Future research should move beyond one-time associations and incorporate repeated measurement, activity context, and multilevel school characteristics to clarify whether the present pattern is primarily relational rather than merely behavioral.

The exploratory parenting models deepen the contextual interpretation. Once father- and mother-related composites were entered, the PAQ coefficient for loneliness attenuated, while father support and father harsh control showed stronger associations. The point is not that physical activity no longer matters; rather, it appears to matter within a relational environment that may either amplify or constrain its social relevance. Because these parenting composites were content-derived and the study was cross-sectional, these findings should be viewed as exploratory and hypothesis-generating rather than as evidence of a mechanism.

### 4.5 Public health implications

For school health practices, the current findings argue against a narrow dosage-only message. Increasing physical activity remains worthwhile because it has broad developmental and health benefits for school-aged youth, even when mental health gains are selective rather than universal (2, 44). However, if the public health goal is to improve psychological wellbeing in minority school settings, intervention design should pay attention to the social architecture of participation: whether the activity is enjoyable, inclusive, peer-connected, and relationally supportive. In this sample, such strategies appear most relevant to loneliness and social connectedness rather than to generalized anxiety or social anxiety more broadly.

### 4.6 Strengths and limitations

This study has several strengths. It focused on an under-researched ethnic minority student population, used a moderately large school-based analytical sample, applied transparent data cleaning rules, and modeled three distinct adverse mental health domains rather than collapsing them into a single undifferentiated total. It also complemented internal consistency estimates with sample-based structural validity support and included robustness checks plus exploratory family-context models. The study also has important limitations. Its cross-sectional design precludes causal inference and does not allow temporal ordering to be established. Reverse causation and bidirectional relationships remain possible. All measures were self-reported; accordingly, the PAQ-like score should be interpreted as an index of habitual activity rather than an objective measure, and the present 8-item instrument should be interpreted more cautiously than a direct administration of the full original PAQ-C / PAQ-A forms (34, 35). In addition, potentially relevant covariates such as sleep, screen time, socioeconomic conditions, family structure, academic stress, and body composition were not available in the final analytical dataset and therefore could not be included in the adjusted models. Residual confounding should therefore be considered when interpreting the findings. The senior secondary subgroup was relatively small, limiting the precision of stage-stratified analyses. Finally, the parenting extension models relied on content-derived composites rather than officially published subscales and should therefore be viewed as exploratory.

## 5 Conclusion

Among Yi students aged 10–18 years in Southwest China, the association between physical activity and mental health varied across outcomes. Higher physical activity was modestly but consistently associated with lower loneliness, whereas the adjusted associations with total anxiety and social anxiety were not robust. These findings should be interpreted as cross-sectional and associational rather than causal. They support a more differentiated interpretation of youth physical activity in which social connectedness and family climate may shape mental health relevance in minority school settings.

## Data Availability

The anonymized data upon which the conclusions of this paper are based will be provided by the corresponding author upon reasonable request, subject to institutional data protection requirements and school-level permission.
